# ﻿First record of *Morimotobathynella* Serban, 2000 (Bathynellacea, Bathynellidae) from subterranean waters of South Korea, with the description of a new species

**DOI:** 10.3897/zookeys.1224.141117

**Published:** 2025-01-22

**Authors:** Su-Jung Ji, Ana Isabel Camacho, Gi-Sik Min

**Affiliations:** 1 Department of Biological Sciences and Bioengineering, Inha University, Incheon 22212, Republic of Korea Inha University Incheon Republic of Korea; 2 Division of Biomedical Research, Korea Research Institute of Bioscience and Biotechnology, Daejeon 34141, Republic of Korea Korea Research Institute of Bioscience and Biotechnology Daejeon Republic of Korea; 3 Museo Nacional de Ciencias Naturales (CSIC), Dpto. Biodiversidad y Biología Evolutiva, Madrid 28006, Spain Museo Nacional de Ciencias Naturales Madrid Spain

**Keywords:** Interstitial hyporheic zone, Korean peninsula, molecular analysis, *Morimotobathynellakoreana* sp. nov., subterranean crustacea, taxonomy

## Abstract

This study describes *Morimotobathynellakoreana***sp. nov.**, the first new species of Bathynellidae family reported in East Asia since 2000, and it presents the first molecular analysis using CO1 and 18S gene sequences. Morphological analysis reveals that the new species and previously known *Morimotobathynella* species uniquely share key characteristics in the male and female thoracopods VIII. However, the presence or absence of the median seta on the antenna exopod, along with the length differences between the four spines in the furca, distinguish the new species from *M.miurai*, 2000. A molecular phylogenetic analysis indicates that the new species has a relatively close relationship to species from the genus *Altainella* in Mongolia and Russia.

## ﻿Introduction

The order Bathynellacea Chappuis, 1915, found exclusively in freshwater subterranean environments, currently comprises over 340 species distributed among three families: Bathynellidae Grobben, 1905, Parabathynellidae Noodt, 1965, and Leptobathynellidae Noodt, 1965 (Camacho et al. 2021). Unlike Parabathynellidae and Leptobathynellidae—with the latter often considered synonymous with the former—the family Bathynellidae is characterized by smaller, more fragile bodies and stable morphological traits (Drewes and Schminke 2011; Camacho 2015; Camacho et al. 2018a). Consequently, there is a lack of described morphospecies and limited taxonomic knowledge of Bathynellidae compared to the Parabathynellidae. Despite their global distribution, Bathynellidae are currently represented by only about 110 known species in 36 genera worldwide (Camacho et al. 2020; Ji et al. 2024; Perina et al. 2024). This family can be distinguished from other families by several morphological characteristics: it has a pair of setae on the dorsal side of the pleotelson, the antennae possess an exopod, and the reproductive appendages in females are relatively less simplified than those in other families (Camacho 2015; Camacho et al. 2018a, 2021).

Research on the family Bathynellidae in East Asia—Korea, China, and Japan—has a relatively short history. Initial studies began in Japan in the mid-20^th^ century and primarily focused on morphological studies using samples collected from wells (Morimoto 1959, 1970a, 1974; Uéno 1952, 1954, 1961). In Korea, research on Bathynellidae started with Morimoto (1970b), who described four species of the genus *Bathynella* Vejdovsky, 1882—*B.rufa*, *B.fodinarum*, *B.minuta*, and *B.uenoi*—from caves and wells. To date, these species remain the only known representatives of this family on the Korean Peninsula. No research has been conducted on Bathynellidae in China to date. Meanwhile, the Bathynellidae species found in East Asia have not been examined using molecular phylogenetic methods, and their phylogenetic placement relies solely on the morphological analysis provided by Serban (2000).

This study describes the first new species of Bathynellidae collected from East Asia since 2000, which was collected in South Korea. The new species was assigned to the genus *Morimotobathynella* Serban, 2000, which previously had only one species, *M.miurai* Serban, 2000, from Japan. In addition, we provide the first molecular study of East Asian Bathynellidae species, presenting a global phylogenetic analysis based on the CO1 and 18S gene sequences obtained from this new species.

## ﻿Materials and methods

### ﻿Sampling and morphological observation

Samples were collected from the interstitial hyporheic zone of Hongcheon-gun, South Korea (Suppl. material 1). For sampling water from the hyporheic zone, a 1 m core was driven into the points using a hammer, and water was collected using a manual pump and filtered using a 50 μm fine-mesh net (Lee and Park 2016). Specimens were immediately preserved in 95% ethanol. Specimen of *Morimotobathynellakoreana* sp. nov. were dissected in glycerol under a stereomicroscope (SZX12, Olympus, Japan). Dissected appendages were mounted using Eukitt® Quick-hardening mounting medium (Sigma-Aldrich, St. Louis, MO, USA) for permanent slide. Observation and drawing were conducted using an optical microscope (DM2500, Leica, Germany). The type materials of the new species examined in this study have been deposited in the collection at the Nakdonggang National Institute of Biological Resources, Korea (**NNIBR**).

### ﻿Molecular phylogenetic analysis

The genomic DNA was extracted from the tissue using the LaboPass™ Tissue Genomic DNA Isolation Kit Mini (Cosmo GENETECH, Seoul, South Korea) according to the manufacturer’s instructions. Amplification by polymerase chain reaction was conducted using the following primer sets: C1-J1718 and C1-J2329 (Simon et al. 1994) for the mitochondrial CO1 gene; 1F, 5R or 3F, 9R (Giribet et al. 1996) for the nuclear 18S gene. The sequences were aligned using ClustalW (Thompson et al. 1994; Larkin et al. 2007) in Geneious Prime (v. 2024.0.2). The intraspecific genetic *p*-distances based on 18S rRNA gene sequences for the family Bathynellidae, including the new species were determined using MEGA X v. 10.1.8 (Kumar et al. 2018).

### ﻿Phylogenetic analysis

Phylogenetic analyses were performed using maximum likelihood (ML) and Bayesian inference (BI) based on the concatenated sequences of mitochondrial CO1 and 18S rRNA genes. Prior to phylogenetic analysis, 18S rRNA sequences were individually trimmed using Gblocks v. 0.91b with default parameters to eliminate poorly aligned positions and divergent regions (Castresana 2000; Talavera and Castresana 2007). The resulting clean alignments of the 18S rRNA and CO1 sequences were concatenated into a single dataset for subsequent analyses. ML analysis was performed using IQ-TREE v. 1.6.8 with ModelFinder to select the best-fit substitution model. The best-fit model selected according to the Bayesian Information Criterion was GTR+F+I+G4 (Kalyaanamoorthy et al. 2017; Huang et al. 2023). Node support values were assessed using ultrafast bootstrap approximation with 1,000 replicates. Prior to the BI analysis, the best-fit nucleotide substitution model was selected using jModelTest v. 2.1.7 software based on the Akaike Information Criterion, and the GTR+I+G model was selected (Darriba et al. 2012). The BI assessment was performed using MrBayes v. 3.2.6 for 1 million generations (Ronquist et al. 2012); the first 30% of the generations were discarded as burn-ins. The final trees were displayed in FigTree v. 1.4.4 and edited using Adobe Illustrator.

## ﻿Results

**Order Bathynellacea Chappuis, 1915**

**Family Bathynellidae Grobben, 1905**

**Subfamily Bathynellinae Grobben, 1905**

### ﻿Genus Morimotobathynella

Taxon classificationAnimaliaBathynellaceaBathynellidae

Serban, 2000

F28BEA69-51EF-5448-98D6-F09169ED29C7

#### Amended generic diagnosis.

Antennule and antenna 7-segmented. ***Pars molaris*** of the mandible formed by two teeth near the ***processus incisivus accessorius*** and two lobe with distal region covered by denticles. Endopod of thoracopods I–VII 4-segmented. Thoracopod I with coxal seta. Male thoracopod VIII with massive protopod; penial region with three formations, an anterior lobe and two formations (inner and outer) like lamella, with the distal part curved towards the median axis of the appendix, that form an “atrium”; robust endopod with elliptical transversal section, basipod without anterior prominence, small anterior lobe. Thoracopod VIII of female with well-developed epipod with only exopod; absent endopod. Uropod with few setation, with true uropodal claws on the endopod. Furcal rami with robust and short spines.

### ﻿ Morimotobathynella koreana

Taxon classificationAnimaliaBathynellaceaBathynellidae

Ji, Camacho & Min, 2024 sp. nov.

91FE1F0B-A552-55F8-8C51-29F3B328BB44

https://zoobank.org/02D78393-4E81-4F71-B3D3-30F02D548C6C

**Figure 1.**
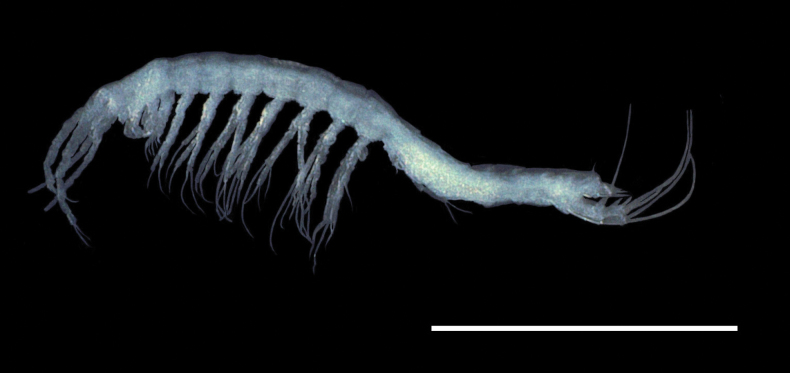
Habitus of *Morimotobathynellakoreana* sp. nov. (female, NNIBRIV136391). Scale bar: 0.5 mm

**Figure 2.**
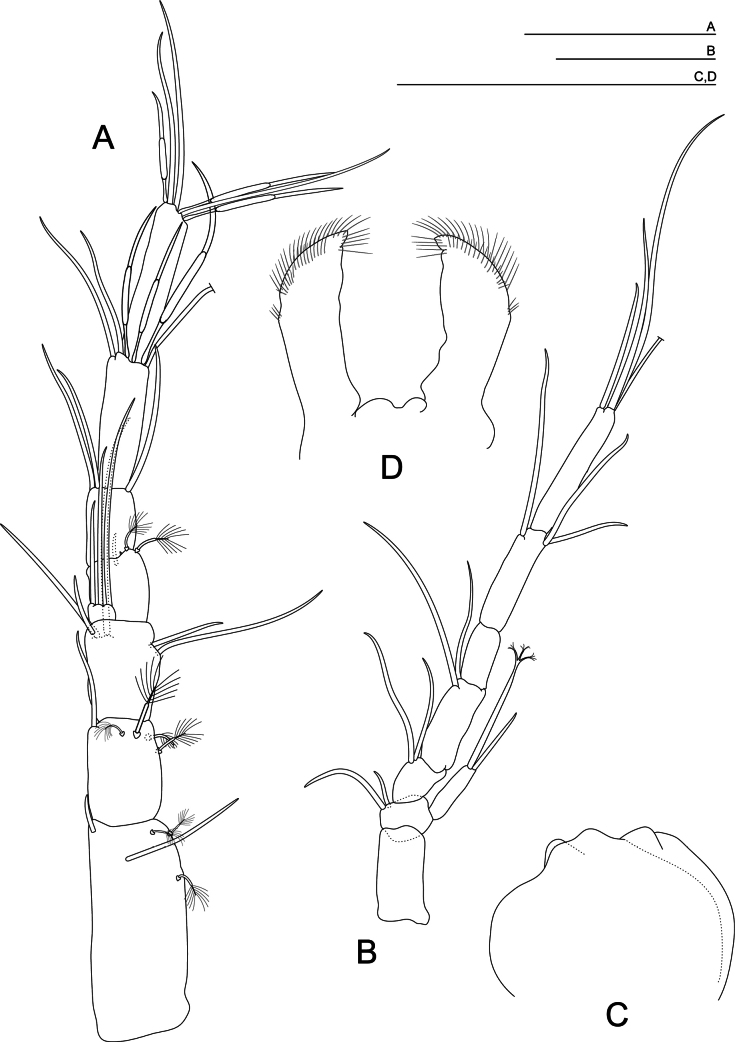
*Morimotobathynellakoreana* sp. nov., holotype male **A** antennule **B** antenna **C** labrum **D** paragnath. Scale bars: 0.05 mm.

**Figure 3.**
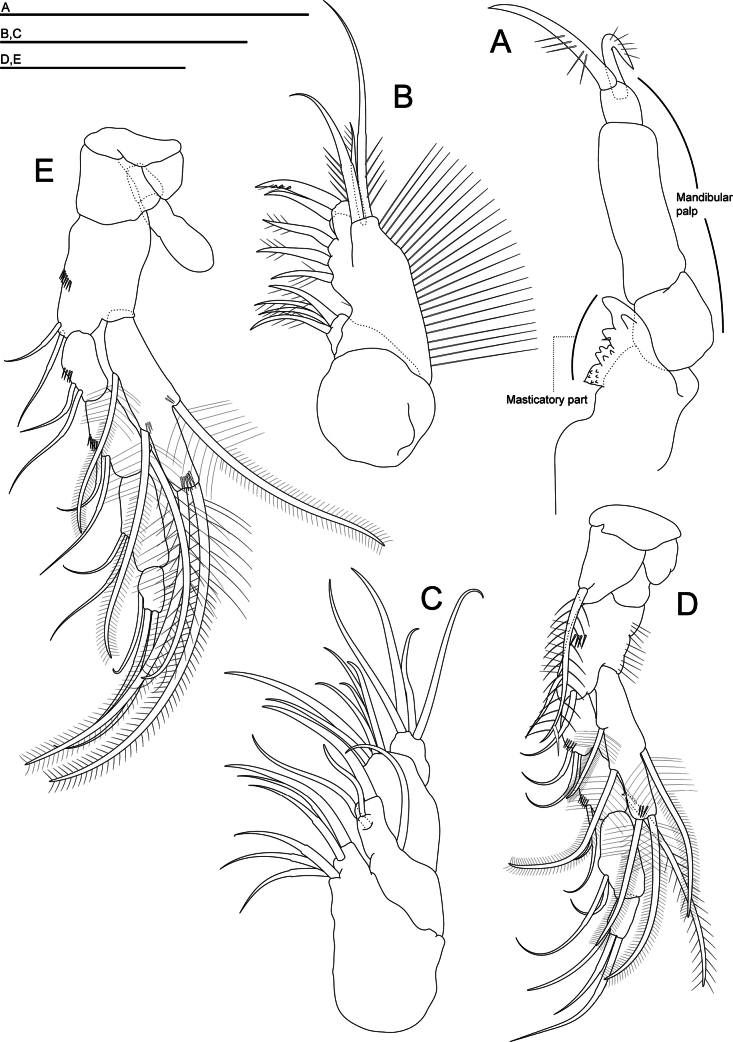
*Morimotobathynellakoreana* sp. nov., holotype male **A** mandible **B** maxillule **C** maxilla **D** thoracopod I **E** thoracopod II. Scale bars: 0.05 mm.

**Figure 4.**
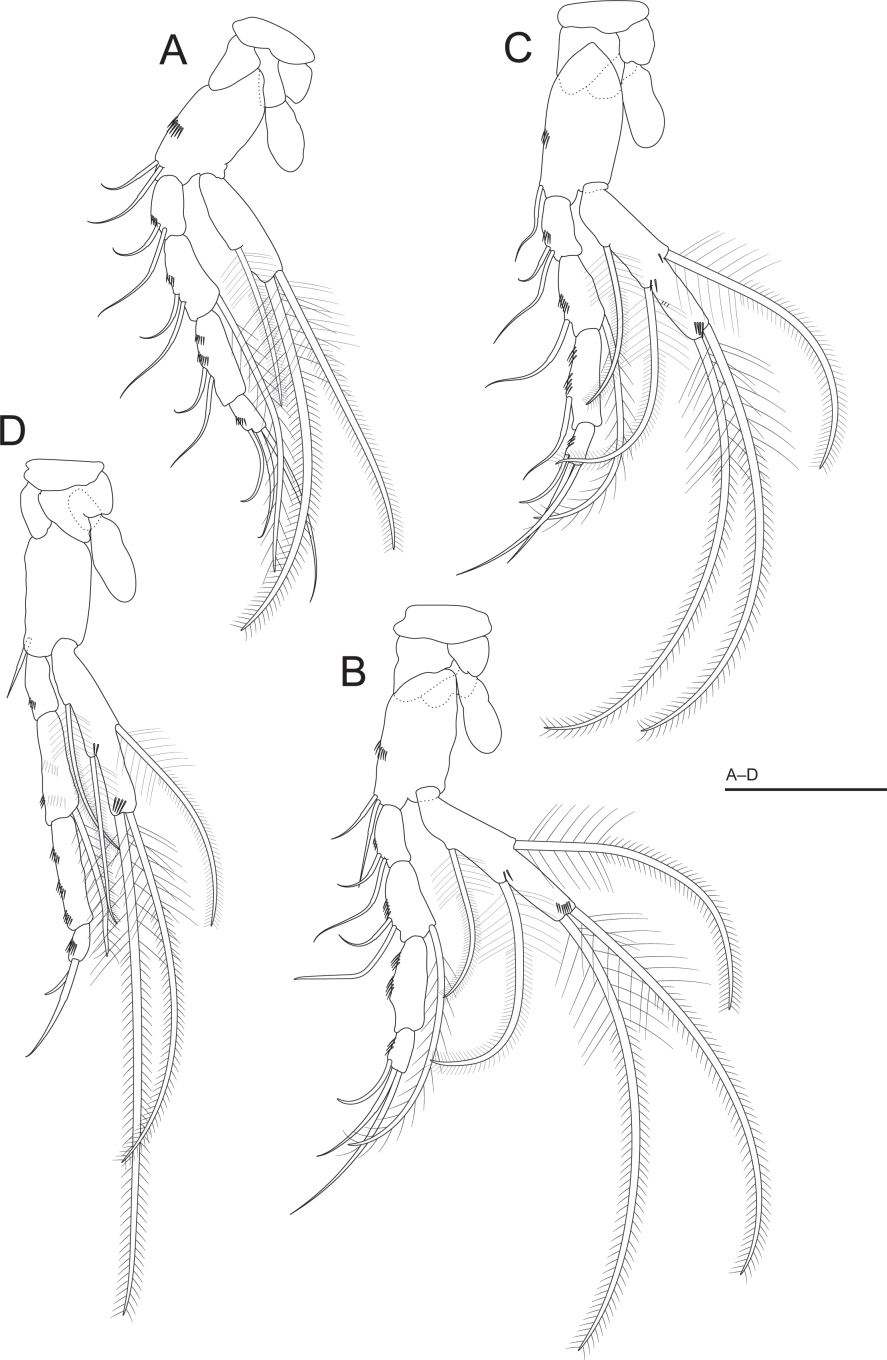
*Morimotobathynellakoreana* sp. nov., holotype male **A** thoracopod III **B** thoracopod IV **C** thoracopod V **D** thoracopod VI. Scale bars: 0.05 mm.

**Figure 5.**
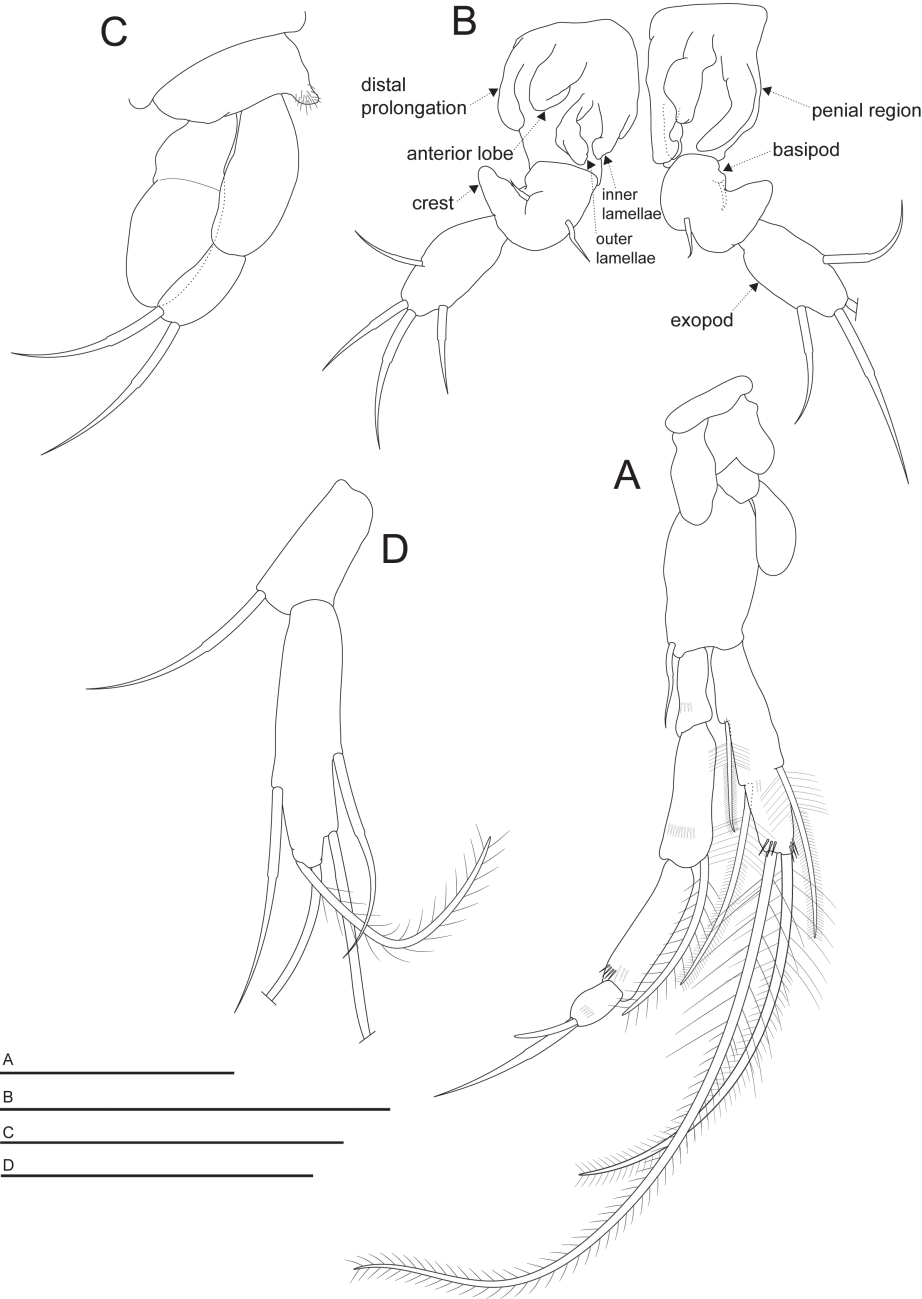
*Morimotobathynellakoreana* sp. nov., (**A, B, D**) holotype male, (**C**) allotype female **A** thoracopod VII **B** thoracopod VIII **C** thoracopod VIII **D** pleopod. Scale bars: 0.05 mm.

**Figure 6.**
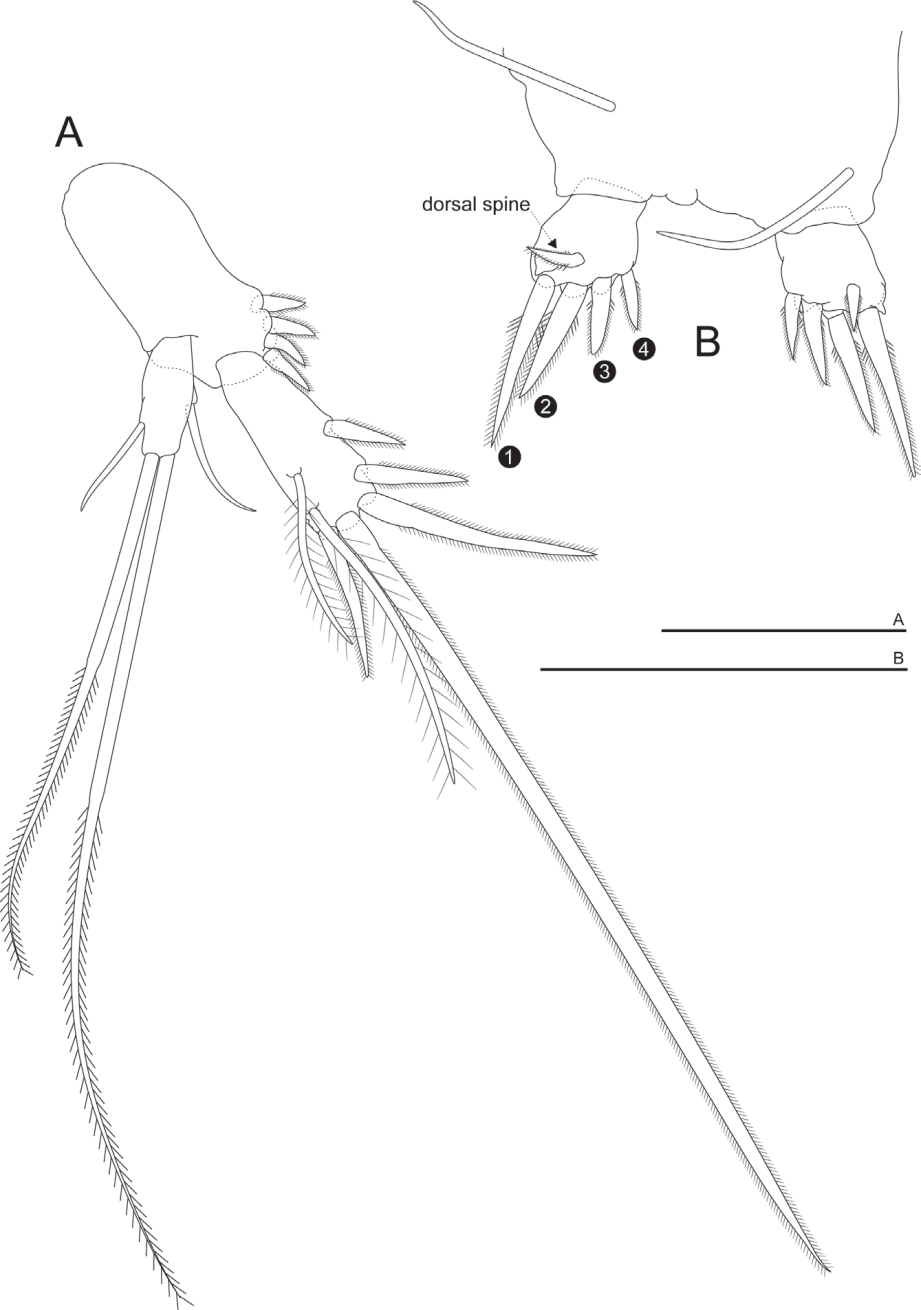
*Morimotobathynellakoreana* n. sp., holotype male **A** uropod **B** pleotelson and furcal rami. Scale bars: 0.05 mm.

Figs 1 , 2 , 3 , 4 , 5 , 6 Korean name: 한국모리모토옛새우(신칭)

#### Type locality.

Hongcheon-gun (37°41'15.82"N, 127°41'0.53"E), South Korea; collected by G.-S. Min, C.-W. Lee, and H.-M. Yang on 4 March 2016.

#### Type materials.

***Holotype***: male (NNIBRIV136387), dissected on six slides. ***Allotype***: female (NNIBRIV136388), dissected on five slides. ***Paratypes***: 3 females (NNIBRIV136389–136391).

#### Diagnosis.

Antennule and antenna 7-segmented; antennule much longer than antenna. Antenna exopod without median seta. Mandible: mandibular palp with three articles; pars molaris with two lobes bearing small denticles distally, lacking a prominent terminal tooth. Endopod of thoracopods I–VII 4-segmented; coxa of thoracopod VII with a strong plumose seta on thoracopod I; sexually dimorphic in thoracopod VIII of males and females. Male thoracopod VIII: massive protopod with penial region forming an “atrium,” inner and outer lamellae curving towards the center; basipod with a rounded crest and one distal seta; exopod elongated with a robust apex and elliptical transversal section; endopod absent. Female thoracopod VIII: coxa with a small protrusion with setules, a very large and well-developed epipod exceeding the basipod length, and an exopod with two equal long setae, lacking an endopod. Uropod: sympod with five spines; endopod with long and strong terminal setae; exopod with four setae, two barbed terminally. Furcal rami with the first spine nearly twice as long as the others.

#### Description.

**Adult male. *Total body*** (Fig. 1) 0.9 mm in length. Cylindrical and elongate body with a similar diameter on thoracic and abdominal articles. Head longer than wide. Pleotelson with one small dorsal seta on each side.

***Antennule*** (Fig. 2A) 7-segmented; the first three articles equal in length to the last four combined; the first article is the longest and the fourth is the smallest; small rectangular inner flagellum; the third article with five smooth setae; the sixth and seventh articles with three aesthetascs of different sizes; antennule much longer than antenna.

***Antenna*** (Fig. 2B) 7-segmented; without a medial seta on exopod; the first, fourth, and sixth articles similar in length; the second and third articles similar in length and the shortest; fifth article small, measuring just over half length of first article; the last article is the longest, 1/3 longer than the first one; setal formula: 0+0/2+0/2+0/2+0/0+0/1+2/4.

***Labrum*** (Fig. 2C). The distal smooth free edge, with central irregular protuberances.

***Paragnaths*** (Fig. 2D) almost rectangular; having setulation on the distal half.

***Mandible*** (Fig. 3A). Palp with three articles, the third article with two claws of different length, the first and second articles rectangular and robust, and the third article small and almost square; masticatory part: incisor process (***pars incisiva***) with two teeth; accessory incisor process (***processus incisivus accessorius***) with one tooth and one tiny spine; molar part (***pars molaris***) with two lobes having small denticles.

***Maxillule*** (Fig. 3B). The proximal endite with four setae, all setulose; the distal endite having five teeth and the distal one with three denticles; three plumose setae of different length on the outer margin.

***Maxilla*** (Fig. 3C) 4-segmented; setal formula 6, 4, 6, and 4.

***Thoracopods I–VII*** (Figs 3D, E, 4A–D, 5A). Well developed; thoracopod I–III (Figs 3D, E, 4A) progressively longer; thoracopod IV–VI (Fig. 4B–D) of similar length; thoracopod VII (Fig. 5A) longer than the others; thoracopod I–VII with epipod a little longer than half the basipod; coxa with long strong plumose seta on thoracopod I; rectangular basipod with two smooth setae on thoracopods I–IV, with only one seta on thoracopod V–VII.

***Exopods of thoracopods I–VII*** (Figs 3D, E, 4A–D, 5A) 1-segmented with barbed setae (two terminal, one dorsal and two ventral) and shorter than endopods of thoracopods I–VII; as long as the first two articles combined in thoracopods I–III and VI–VII, reaching the middle of the third endopodal article in thoracopods IV and V.

***Endopods thoracopods I–VII*** (Figs 3D, E, 4A–D, 5A) 4-segmented; the first two articles similar in length in thoracopod I; second article longer than the first article, equal in length to the third article in thoracopods II and VII; the second article very long in thoracopods VI and VII; the fourth article small in all thoracopods. Setal formula of endopods (the number of setae on basipod in brackets):

Thoracopod I: (2) 2+0/2+1/2+0/3

Thoracopods II, III: (2) 2+0/2+1/2+0/3

Thoracopod IV: (2) 2+0/2+1/2+0/3

Thoracopod V: (1) 2+0/1+1/1+0/3

Thoracopods VI, VII: (1) 0+0/0+1/0+0/2(1)

***Thoracopod VIII*** (Fig. 5B) with a massive protopod; penial region with the distal prolongation similar in size to the three other formations: an anterior lobe and two formations (inner and outer) resembling lamellae rather than lobes, with the distal part curved towards the median axis of the appendix, forming an “atrium”; basipod with a rounded crest with a small seta at the base; robust exopod with elliptic transversal section and four setae; endopod absent.

***Pleopods*** (Fig. 5D) 2-segmented; the first article with very long smooth seta; the second article with five setae: four smooth setae and one barbed seta of different lengths.

***Uropods*** (Fig. 6A). Sympod 50% longer than wide and as long as the endopod, with four equal distal spines; endopod almost 50% longer than exopod, with three strong spines, the distal one being two times longer than second which is longer than the first, and one fourth terminal spine thinner than the other three, and on distal end there are a long and strong special seta, and two plumose setae located dorso-laterally; exopod with four setae, two terminal barbed, of different length, and two short medial setae.

***Pleotelson*** (Fig. 6B) with one barbed dorsal seta on each side near the base of furca, each extending beyond furcal rami

***Furcal rami*** (Fig. 6B) almost square, bearing five spines; the first spine almost twice as long as the fourth and slightly longer than the second, which itself a bit longer than the third; the fourth spine about the same length as the dorsal spine.

**Adult female.** The female is similar to the male in all its features except for thoracopod VIII.

***Thoracopod VIII*** (Fig. 5C). Coxa with a small protrusion with setules; having a very well-developed epipod exceeding the length of the basipod, reaches the distal end of the exopod; with only one ramus, the exopod with two equal long setae, absent endopod.

#### Morphological remarks.

Morphological comparisons of the new species with the three subfamilies within Bathynellidae, as well as comparisons between the two species of the genus *Morimotobathynella*, are listed in the tables (Suppl. material 2: table S1, Table 1). This section presents a detailed examination of the morphological characteristics of the appendages of these two *Morimotobathynella* species.

**Table 1.** Morphological differences among *Morimotobathynellamiurai* Serban, 2000 and *M.koreana* sp. nov.

	*M.miurai*	*M.koreana* sp. nov.
Country	Japan	South Korea
**Antennule**		
Aesthetacs on sixth article	3	3
Aesthetacs on seventh	3	3
**Antenna**		
Medial seta on exopod	Present	Absent
Setal formula	0+0/2+0/2+0/2+0/0+0/0+0/5	0+0/2+0/2+0/2+0/0+0/1+2/4
AI vs. AII	AI < AII	AI > AII
**Mandible**		
Teeth	5+small lobe with denticles	7+denticles
**Paragnath**	with setules and strong tooth	with setules
**Maxillule**		
Setules on outer margin	Absent	Present
**Maxilla**		
Setal formula	—	6, 4, 6, 4
**Thoracopod I-VII**		
Epipod on thoracopod I	Absent	Absent
Number of setae on thoracopods I-VII exopod	5-5-5-5-5-5-5	5-5-5-5-5-5-5
(Basipod setae) Setal formula of thoracopod I-VII endopod	(4) 4+0/3+1/3+0/4	(2) 2+0/2+1/2+0/3
	(3) 3+0/3+1/3+0/4	(2) 2+0/2+1/2+0/3
	(3) 3+0/3+1/3+0/4	(2) 2+0/2+1/2+0/3
	(3) 3+0/3+1/2+0/3	(2) 2+0/2+1/2+0/3
	(1) 1+0/0+1/0+0/2	(1) 2+0/1+1/1+0/3
	(1) 1+0/0+1/0+0/2	(1) 0+0/0+1/0+0/2(1)
	(1) 1+0/0+1/0+0/2	(1) 0+0/0+1/0+0/2(1)
**Thoracopod VIII of female**		
Coxa	With setulated protuberance on inner margin	With setulated protuberance on inner margin
Basipod	With 2 setae on inner margin	Without setae
Endopod	absent	Absent
Exopod	With 2 terminal setae of similar length	With 2 terminal setae of similar length
Epipod	Exceeding the length of basipod and exopod combined	Similar in length to the basipod and exopod combined
**Thoracopod VIII of male**		
Distal prolongation	Present	Present
Anterior lobe	Present	Present
Inner lamella vs. outer lamella	Inner < outer, two lamellae completely closed, forming a small inner space	Inner = outer, two lobes not completely closed, Forming a small inner space.
Basipod axis	Vertical	Inclined
Crest (anterior prominence) on basipod	Absent	Blunt-tipped conical shape
Endopod	Present; fused with basipod	Absent
Exopod	Like exopod of thoracopods	Like exopod of thoracopods
**Pleopod**		
First article	1 seta	1 seta
Second article	3 setae	5 setae
**Uropod**		
Number of spines on sympod	4	4
Sympod vs. endopod	Sympod > endopod	Sympod = endopod
Length of endopod	Slightly longer than exopod	Twice longer than exopod
**Furca**		
Spines length comparison	Dorsal spine <4<1<3<2	Dorsal spine = 4<3<2<1
Dorsal spines	Very small	Similar to the fourth spine
Dorsal seta of pleotelson	Absent	Exceeding the furcal rami

1. The size proportions of antennule articles differ between the two species. In the new species, the first three articles are approximately the same length as the last four articles, with the first being the longest and the fourth being the shortest. In *M.miurai*, the combined lengths of the first three articles were greater than those of the last four articles. In addition, the second and third articles are twice as long as the fourth and fifth articles, which are the shortest and equally long, respectively.
2. In the new species, the length of antennule is greater than that of antenna, whereas in *M.miurai*, antennule is shorter than antenna.
3. In both species, the longest article is the last article, followed by the first, fourth, and sixth articles of the same length. The second, third, and fifth articles are the shortest and very similar in length in the new species. In contrast, in *M.miurai*, the fifth article is twice as long as the second and third articles, which are similar in size and are the smallest.
4. In the new species, the pars molaris of the mandible does not have a terminal tooth larger than the denticles on the two lobes, as observed in *M.miurai*.
5. The new species has dense setules on the outer margin of the maxillule, which are absent in *M.miurai*.
6. The setal formula of the thoracopods differs between the two species (see Discussion and Table 1). However, we cannot compare the proportions of the articles because the thoracopods were not illustrated in the original description of *M.miurai*.
7. Thoracopod VIII in males differs significantly between the two species; *M.miurai* has an endopod with two setae, that are absent in the new species.
8. In thoracopod VIII of female, *M.miurai* has a large epipod that extends beyond the exopod, whereas in the new species, the epipod does not exceed the length of the exopod. *Morimotobathynellamiurai* has two setae on the basipod, that are absent in the new species.
9. In the uropod, *M.miurai* has four spines on the sympod that are similar in pairs, with the two distal spines being longer than the two proximal spines. In the new species, all four spines on the sympod are similar in length and shorter than those of the type species of the genus. Additionally, in *M.miurai*, the exopod and endopod are equal in length, whereas in the new species, the endopod is twice as long as the exopod.
10. In the furcal rami, the longest spine in *M.miurai* is the second, which has nearly the same length as the third, while the first and fourth spines are similar in size and slightly smaller, and the dorsal spine is very small. In the new species, the longest spine is the first, with the spines gradually decreasing in length; the fourth is one-third the length of the first and similar in size to the dorsal spine.

#### Etymology.

The specific epithet “*koreana*” is derived from South Korea, the country where the new species was discovered.

### ﻿ Morimotobathynella

Taxon classificationAnimaliaBathynellaceaBathynellidae

sp.

539623AD-D3F1-51AE-9353-0AC5C45AA879

#### Material examined.

Pocheon-si (38°6'56.21"N, 127°15'46.38"E), South Korea. Collected by S. -J. Ji and C. -W. Lee on 31 May 2020.

#### Remarks.

This specimen was included in the phylogenetic analysis to mitigate potential long-branch attraction issues that could affect the phylogenetic placement of *M.koreana* sp. nov. The inclusion of this closely related species provides a more robust phylogenetic framework for the genus *Morimotobathynella* in East Asia. Although a detailed morphological examination suggests that this specimen represents another potentially new species, a formal description requires additional material.

##### ﻿CO1 and 18S rRNA gene sequencing

In this study, 597 bp of CO1 (PQ790059–PQ790061) and 1,713 bp of 18S sequences (PQ789943, PQ789944) were obtained from *M.koreana* sp. nov. The three CO1 and two 18S sequences obtained from *M.koreana* sp. nov. showed no intraspecific variation. Additionally, 597 bp of CO1 (PQ790062) and 1,210 bp of 18S sequences (PQ789945) were obtained from *Morimotobathynella* sp. collected in Pocheon, South Korea. Genetic divergence analysis revealed that the two Korean *Morimotobathynella* species had *p*-distances of 10.4% for CO1 and 1.2% for 18S sequences.

##### ﻿Phylogenetic analysis

**Figure 7.**
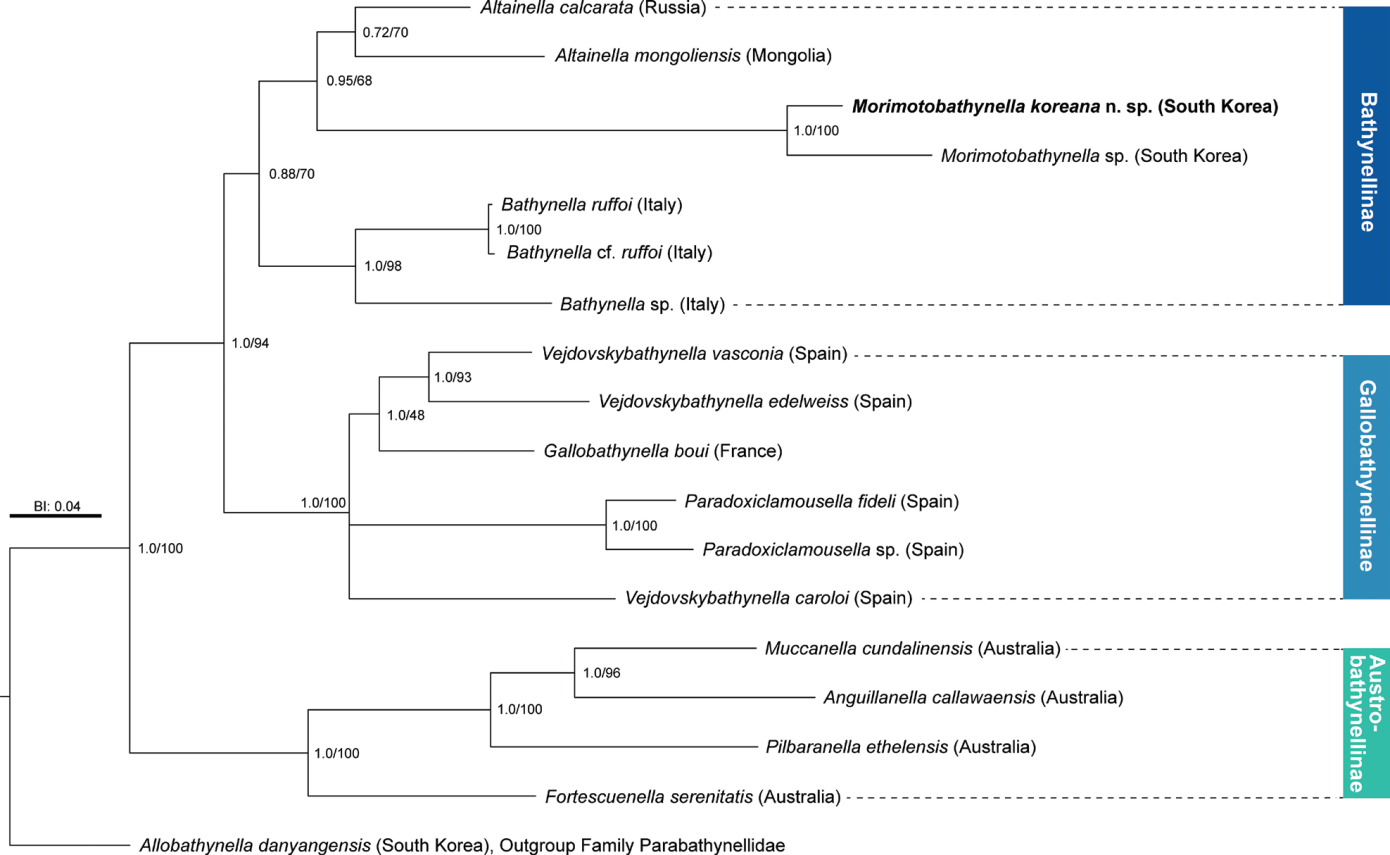
Maximum likelihood and Bayesian inference analyses based on nuclear 18S and mitochondrial CO1 sequences (1,002 bp). Numbers on nodes represent bootstrap values for maximum likelihood and Bayesian posterior probabilities.

ML and BI analyses were performed using a concatenated 1,002 bp dataset comprising CO1 (615 bp) and 18S rRNA (387 bp) sequences from the new species and 18 other Bathynellidae species available in GenBank (Fig. 7, Suppl. material 2: table S2). *Allobathynelladanyangensis* Ji & Min, 2023 from South Korea (Family Parabathynellidae) was used as outgroup, and the analysis confirmed the monophyly of Bathynellidae. Both the ML and BI analyses produced congruent tree topologies. Our results also support the separation of three distinct subfamilies within Bathynellidae, corroborating previous studies (Camacho et al. 2013, 2020). Phylogenetic trees revealed that *M.koreana* sp. nov. and *Morimotobathynella* sp. from South Korea formed a distinct monophyletic lineage within the subfamily Bathynellinae, with strong support (bootstrap support: 100%, posterior probability: 1.0). They showed a close relationship with species of the genus *Altainella* from Mongolia and Russia (bootstrap support: 68%, posterior probability: 0.95). This phylogenetic relationship may suggest a historical biogeographic connection among bathynellid taxa in Northeast Asia.

## ﻿Discussion

Six genera within the family Bathynellidae have been identified in East Asia: *Bathynella* Vejdovsky, 1882; *Uenobathynella* Serban, 2000; *Parauenobathynella* Serban, 2000; *Nihobathynella* Serban, 2000; *Paradoxibathynella* Serban, 2000; and *Morimotobathynella* Serban, 2000 (Morimoto 1970b; Serban 2000). Of these, five genera, except *Bathynella*, were all found and recorded only in Japan (Serban 2000). The only known species belonging to the genus *Morimotobathynella*, *M.miurai* Serban, 2000, and *M.koreana* sp. nov., share the following phylogenetically significant morphological characteristics: the female thoracopod VIII has only the exopod without the endopod; the female thoracopod VIII has a small projection with ctenidia on the inner margin and a large epipod; and the inner and outer lamellae located on the inner side of the penial region of the male thoracopod VIII curve towards the center, forming a narrow space—referred to as the “atrium” (see Description and Fig. 5). Based on this morphological evidence, we assigned a new species from South Korea to the genus *Morimotobathynella*. Serban’s (2000) description of *M.miurai* is relatively detailed, and the morphological comparison of the two species within the genus *Morimotobathynella*, presented in Table 1, confirms that they are clearly distinct despite sharing evolutionary important characteristics. However, no molecular support currently exists to distinguish species within the family Bathynellidae from East Asia, including the genus *Morimotobathynella*.

The molecular phylogenetic analysis of the Bathynellidae revealed several key findings (Fig. 7). Our results placed the two *Morimotobathynella* species collected from South Korea within the subfamily Bathynellinae, one of the three recognized subfamilies within Bathynellidae. Despite its relatively long branch, the genus *Morimotobathynella* showed a closer phylogenetic relationship with the Asian clade represented by *Altainella* species from Mongolia and Russia than with the European or Australian taxa (Fig. 7). Meanwhile, phylogenetic analyses of the families Bathynellidae and Parabathynellidae, revealed similar biogeographic patterns, with separation into Eurasian and Australian clades (Camacho et al. 2018b; Ji and Min 2023a, 2023b). These results indicate that the two families, although independently evolved, may have responded similarly to shared paleogeographic events such as tectonic events or faults, owing to their exclusive presence in continental groundwater environments. This phylogeographical aspect has been relatively well documented in the family Parabathynellidae but remains understudied in Bathynellidae.

Serban (2000) assigned the genus *Morimotobathynella* to the subfamily Bathynellinae, which is consistent with the results of the molecular phylogenetic analysis presented in this study. However, morphological examination revealed that *M.koreana* sp. nov. did not match the currently recognized diagnostic characteristics of the three subfamilies (Suppl. material 2: table S1). The inability to place the new species within existing subfamilies based on morphology suggests that the current morphological diagnosis for subfamily classification within Bathynellidae may be insufficient or require revision. While this study points to the need for refinement of morphological diagnoses to better reflect phylogenetic relationships, such revisions require broader comparative studies across diverse bathynellid taxa. To resolve this issue, prioritizing morphological and molecular phylogenetic studies on bathynellid samples from underrepresented regions, such as Asia, South Africa, and South America, is needed.

Hereby, five species within the family Bathynellidae are present in South Korea, including the newly described *M.koreana* sp. nov. and four species of the genus *Bathynella* recorded in 1970 (Morimoto 1970b). As noted in several studies (Camacho et al. 2018a, 2020; Perina et al. 2018), a taxonomic revision and possible reassignment to new genera of these *Bathynella* species may be necessary, although this falls outside the scope of the current research. Considering that 40 species of the family Parabathynellidae, another well-known group of Bathynellacea in South Korea, have been documented, and given their similar evolutionary histories in subterranean freshwater environments, it is likely that the true diversity of Bathynellidae in South Korea is much higher than that currently recognized.

This study is significant because it presents a starting point for new research on Bathynellidae in the East Asian region by describing a newly discovered Bathynellidae species in South Korea through morphological and molecular analyses. Future research should focus on conducting comprehensive field surveys, detailed morphological assessments, and molecular phylogenetic analyses of the family Bathynellidae in this region to uncover hidden species diversity and enhance our understanding of their evolutionary and biogeographical patterns on a global scale.

## Supplementary Material

XML Treatment for Morimotobathynella

XML Treatment for Morimotobathynella koreana

XML Treatment for Morimotobathynella

## ﻿Additional information

### Author ORCIDs

Su-Jung Ji https://orcid.org/0000-0003-3132-2021

Ana Isabel Camacho https://orcid.org/0000-0003-0596-7678

Gi-Sik Min https://orcid.org/0000-0003-2739-3978

### Data availability

All of the data that support the findings of this study are available in the main text or Supplementary Information.

## Supplementary materials

Supplementary material 1 Distribution map of the two species of *Morimotobathynella* Serban, 2000, and photographs of interstitial groundwater sampling in the type locality of *M.koreana* sp. nov. Data type tif https://binary.pensoft.net/file/1229460This dataset is made available under the Open Database License (http://opendatacommons.org/licenses/odbl/1.0/). The Open Database License (ODbL) is a license agreement intended to allow users to freely share, modify, and use this Dataset while maintaining this same freedom for others, provided that the original source and author(s) are credited.Su-Jung Ji, Ana Isabel Camacho, Gi-Sik Min

Supplementary material 2 Aditional information Data type xlsx Explanation note **table S1.** Differences amongst the three subfamilies and Korean new species of the family Bathynellidae (modified from Serban, 1989; Camacho et al. 2018b). **table S2.** CO1 and 18S GenBank numbers used in the molecular phylogenetic study. Bold type indicates sequences newly generated in this study. https://binary.pensoft.net/file/1229461This dataset is made available under the Open Database License (http://opendatacommons.org/licenses/odbl/1.0/). The Open Database License (ODbL) is a license agreement intended to allow users to freely share, modify, and use this Dataset while maintaining this same freedom for others, provided that the original source and author(s) are credited.Su-Jung Ji, Ana Isabel Camacho, Gi-Sik Min
